# Plant-derived bioactives in depression-related and antidepressant-associated sexual dysfunction: a narrative review

**DOI:** 10.3389/fnut.2026.1894055

**Published:** 2026-07-31

**Authors:** Xue Liu, Zhirui Sun, Jia Gao, Chang-Yuan Fu, Zhaoxu Yang

**Affiliations:** 1 Department of Andrology, Jiangsu Province Hospital of Chinese Medicine, Affiliated Hospital of Nanjing University of Chinese Medicine, Nanjing, China; 2 Chengdu University of Traditional Chinese Medicine, Chengdu, China; 3 Karamay Hospital of Integrated Chinese and Western Medicine, Karamay, China

**Keywords:** antidepressant-associated sexual dysfunction, depression, Maca (*Lepidium meyenii*), nitric oxide, plant-derived bioactives, saffron (*Crocus sativus*)

## Abstract

Sexual dysfunction related to depression and antidepressant treatment is clinically important yet underrecognized, affecting adherence, intimate functioning and quality of life. Depression can impair desire, arousal, orgasm and satisfaction before pharmacotherapy, while serotonergic antidepressants may further disturb these domains during treatment, complicating causal attribution and underscoring the need to distinguish depression-related sexual dysfunction from antidepressant-associated sexual dysfunction (AISD). Plant-derived bioactives have attracted interest because several engage systems relevant to both mood and sexual response, including monoaminergic, inflammatory/oxidative, HPA-axis, autonomic and nitric oxide-mediated vascular pathways. We conducted a narrative review based on targeted searches of PubMed/MEDLINE, Web of Science and Scopus through June 2026, synthesizing mechanistic and clinical evidence for selected candidates by phenotype specificity to separate direct AISD studies from adjacent clinical or preclinical evidence and mechanistic plausibility. Saffron provides the most coherent AISD-specific signal, supported by small randomized trials in women and men with fluoxetine-associated sexual dysfunction. Maca shows a narrower signal in female SSRI/SNRI-associated sexual dysfunction, particularly in postmenopausal women. Rosa damascena remains preliminary, whereas *Ginkgo biloba* shows inconsistent controlled evidence and interaction concerns. *Panax ginseng*, pomegranate and dietary nitrates are mechanistically plausible through endothelial and nitric oxide pathways but lack direct AISD evidence. Overall, current evidence supports mechanistic plausibility and preliminary phenotype-specific signals rather than established efficacy. Plant-derived bioactives are best positioned as investigational adjunctive nutritional modulators within evidence-based psychiatric and sexual-medicine care, pending larger trials with standardized preparations, validated sexual-function outcomes, sex-specific analyses and mechanism-oriented biomarkers.

## 1 Introduction

Depression-related and antidepressant-associated sexual dysfunction is a clinically important but often underrecognized challenge at the intersection of psychiatry, sexual medicine and nutritional biology (1). Major depressive disorder can impair sexual desire, arousal, orgasm and overall satisfaction before pharmacological treatment is initiated (2, 3). Serotonergic antidepressants, particularly selective serotonin reuptake inhibitors (SSRIs) and serotonin-norepinephrine reuptake inhibitors (SNRIs), may further contribute to disturbances across these same domains during treatment (4–6). This overlap complicates causal attribution in clinical practice (1). Sexual dysfunction in patients with depression may reflect depressive anhedonia, neuroendocrine stress responses, central monoaminergic regulation, peripheral vascular function and medication-related effects rather than a single isolated adverse drug effect (7–9).

Epidemiological data indicate that the clinical burden is substantial. Antidepressant-associated sexual dysfunction (AISD) has been reported in approximately 30–50% of SSRI-treated patients in conservative estimates, with higher rates when sexual symptoms are actively assessed (4, 5, 10, 11). A recent systematic review and meta-analysis of randomized controlled trials reported that SSRI exposure was associated with a more than threefold higher risk of orgasmic dysfunction and reduced sexual satisfaction compared with placebo (4). Sexual dysfunction is also common in treatment-naive major depressive disorder, affecting approximately 40–50% of patients before antidepressant exposure (2). These multidomain symptoms can contribute to medication non-adherence, premature treatment discontinuation and relapse, while also impairing intimate relationships and quality of life (12–15). Current management strategies, including dose adjustment, antidepressant switching, scheduled drug holidays and adjunctive pharmacotherapy with bupropion or phosphodiesterase type 5 (PDE5) inhibitors, may benefit selected patients but remain limited by relapse risk, incomplete response, tolerability concerns and patient acceptability (12, 16–18). Current strategies may not address all relevant mechanisms that contribute to the multidomain symptom pattern (16).

An important challenge in this field is that depression-related sexual dysfunction and AISD are clinically intertwined and can be difficult to distinguish without documenting the timing of symptom onset relative to antidepressant exposure (1, 19). Human sexual response requires the integration of central motivational circuitry, neuroendocrine regulation, autonomic output and peripheral vascular function (7, 8, 20). Depression may disrupt this integrated system through impaired mesolimbic reward processing, hypothalamic–pituitary–adrenal (HPA) axis activation, systemic inflammatory tone, autonomic imbalance and endothelial dysfunction (9, 21, 22). Serotonergic antidepressants may improve affective symptoms while also increasing inhibitory serotonergic tone over libido, orgasm and genital arousal, partly through downstream effects on dopaminergic and nitric oxide (NO)-mediated pathways (8, 23, 24). This convergence helps explain why the resulting sexual phenotype is often multidomain and only partly responsive to single-target pharmacological approaches (12, 16). Throughout this review, depression-related sexual dysfunction denotes symptoms present before antidepressant exposure, whereas AISD denotes symptoms that emerge or worsen after serotonergic treatment is initiated. Because the two phenotypes may involve partly shared biological pathways but differ in the timing of symptom onset relative to pharmacotherapy, they are distinguished here primarily by this temporal criterion, and studies are interpreted accordingly.

Against this mechanistic background, plant-derived bioactives have attracted research interest (16, 25). Their relevance does not arise from botanical origin alone, but from their potential to influence multiple nutrition-sensitive systems, including monoaminergic signaling, oxidative stress, inflammatory tone, HPA-axis regulation and endothelial NO bioavailability (26–28). Plant-derived bioactives may complement existing approaches by engaging nutrition-sensitive pathways that are also relevant to mood regulation, vascular function and sexual response (27, 29, 30). However, the current evidence is fragmented (16, 31). Some compounds have been evaluated directly in AISD cohorts, whereas others are supported mainly by mechanistic extrapolation from adjacent fields, including depression, cardiometabolic erectile dysfunction or general reproductive health (32–34). The field would benefit from a clearer evidence map linking specific plant-derived candidates to relevant mechanisms, clinical phenotypes and evidence boundaries, distinguishing direct clinical evidence from indirect mechanistic plausibility (16, 31).

This narrative review synthesizes current evidence on plant-derived bioactives in depression-related and antidepressant-associated sexual dysfunction, with emphasis on neuroendocrine and vascular mechanisms. We summarize the biological pathways linking major depressive disorder, serotonergic antidepressant exposure and sexual response, and evaluate how selected plant-derived candidates may interact with these pathways. Particular attention is given to serotonergic, dopaminergic, oxytocinergic, autonomic, HPA-axis and NO-related vascular mechanisms. We also distinguish phenotype-specific findings in AISD from indirect mechanistic evidence and address safety, interaction risks and trial-design considerations. Throughout, plant-derived bioactives are positioned as potential adjunctive nutritional modulators of biological pathways relevant to sexual function, not as replacements for evidence-based psychiatric care or established sexual-medicine interventions. Persistent post-discontinuation sexual dysfunction, including post-SSRI sexual dysfunction (PSSD), involves distinct diagnostic, mechanistic and evidentiary challenges and is outside the present scope.

## 2 Methods

This narrative review was informed by targeted literature searches. We searched PubMed/MEDLINE, Web of Science and Scopus for English-language studies on plant-derived bioactives and sexual function in the context of depression and antidepressant treatment, and supplemented this by screening the reference lists of relevant systematic reviews, meta-analyses, randomized controlled trials and mechanistic studies. Searches were conducted from database inception and last updated in June 2026. Search terms combined the exposure and clinical context (antidepressant, SSRI, SNRI, selective serotonin reuptake inhibitor, serotonin-norepinephrine reuptake inhibitor, major depressive disorder, depression) with sexual-outcome terms (sexual dysfunction, libido, sexual desire, arousal, orgasm, ejaculation, lubrication, erectile function, Female Sexual Function Index, International Index of Erectile Function) and candidate and mechanism keywords (plant-derived bioactives, phytochemicals, saffron, *Crocus sativus*, crocin, maca, *Lepidium meyenii*, Rosa damascena, *Ginkgo biloba*, *Panax ginseng*, ginsenosides, pomegranate, dietary nitrate, beetroot, flavonoids, polyphenols, serotonergic, dopaminergic, oxytocin, nitric oxide, endothelial function, HPA axis, and autonomic regulation). The scope was organized around three interacting neurobiological modules relevant to sexual response: serotonergic and dopaminergic regulation; oxytocinergic, autonomic and HPA-axis regulation; and nitric oxide-dependent vascular function. Evidence was synthesized thematically, prioritizing controlled human evidence in depression-related and antidepressant-associated sexual dysfunction where available; adjacent evidence, including general erectile-dysfunction trials, healthy-adult mood, sleep or vascular studies, and preclinical or mechanistic work, was cited selectively to support biological plausibility and to clarify the boundaries of the evidence. Given substantial heterogeneity in populations, preparations, doses, outcomes and mechanistic platforms, no quantitative meta-analysis was performed.

## 3 Neurobiological mechanisms linking depression, antidepressants and sexual response

Sexual response in both sexes arises from the integration of central motivational drive, neuroendocrine regulation, autonomic balance and peripheral vascular output (7, 8, 20). Depression and serotonergic antidepressant exposure can disrupt this integration at several points along the same biological axis (1, 4). The mechanistic literature converges on three interrelated modules that help explain the multidomain symptom pattern of depression-related and antidepressant-associated sexual dysfunction and provide a biological rationale for selected plant-derived bioactives: serotonergic and dopaminergic regulation, oxytocinergic and autonomic regulation together with HPA-axis activity, and NO-mediated vascular response (7, 9, 24). These modules are considered separately below, although *in vivo* they operate as an interconnected network rather than as independent pathways.

### 3.1 Serotonergic and dopaminergic regulation

Monoaminergic regulation occupies a central position in the pathophysiology of both depression and antidepressant-associated sexual dysfunction (35, 36). Serotonergic neurons originating in the raphe nuclei project widely to the hypothalamus, limbic regions, prefrontal cortex and spinal cord, where they modulate mood, motivation, autonomic outflow and descending control of sexual reflexes (8, 37). In depression, serotonergic neurotransmission is altered in ways that contribute to affective symptoms and changes in sexual desire and response (38). Antidepressant treatments that enhance serotonergic tone, including SSRIs and SNRIs, can relieve depressive symptoms but may also amplify inhibitory serotonergic effects on sexual function (35, 36). The resulting phenotype commonly includes reduced libido, delayed or absent orgasm, ejaculatory disturbance, impaired arousal and diminished genital sensation (4, 5, 39).

The sexual effects of serotonin depend on receptor subtype, anatomical location and treatment context (36, 40). Stimulation of 5-HT2A and 5-HT2C receptors generally inhibits sexual arousal and orgasm, while 5-HT1A receptor activation can facilitate sexual behavior through postsynaptic actions in hypothalamic and spinal regions (8, 41). 5-HT1B receptor activation has also been implicated in delayed ejaculation, consistent with the use of SSRIs as off-label treatment for premature ejaculation (42). These receptor-level effects are not uniform across brain regions or across acute and chronic antidepressant exposure (36, 43). The net clinical effect of SSRI treatment therefore reflects the integrated action of several serotonergic receptor systems rather than a single inhibitory mechanism (36, 44).

Dopaminergic signaling is a major pro-sexual neurotransmitter system, particularly for the motivational and reward-related dimensions of sexual behavior (8, 45). Mesolimbic dopamine projections from the ventral tegmental area to the nucleus accumbens encode the incentive value of sexual stimuli, while dopaminergic signaling in the medial preoptic area and paraventricular nucleus of the hypothalamus contributes to arousal and genital response (8, 46). In men, dopaminergic activation supports penile erection and sexual approach behavior, with D2-like receptor activity playing an important role (8). In women, dopaminergic reward signaling is implicated in sexual desire, subjective arousal and orgasmic response, although the human neuroimaging and pharmacological evidence base remains less extensive than in men (47, 48).

The interaction between serotonergic and dopaminergic systems provides an important mechanism for AISD (8, 23). Elevated serotonergic activity, particularly through 5-HT2 receptor activation, can suppress mesolimbic dopamine release and reduce NO-mediated genital responses, linking SSRI exposure to motivational and vascular components of sexual response (8, 23). Depression is independently associated with blunted reward sensitivity and reduced dopaminergic responsiveness in mesolimbic circuits, contributing to anhedonia, reduced motivation and diminished sexual interest (38, 47). The clinical relevance of this pathway is supported by the lower rates of sexual dysfunction observed with bupropion, a norepinephrine-dopamine reuptake inhibitor, and by its use as an adjunctive option for SSRI-associated sexual side effects (12, 17, 49). A systematic review and meta-analysis of pharmacological treatment for antidepressant-associated sexual dysfunction in women identified bupropion as one of the more consistent options across several domains, while emphasizing low overall certainty of evidence (16). This monoaminergic framework provides a mechanistic anchor for considering plant-derived bioactives that may influence serotonergic, dopaminergic or stress-related signaling (8).

### 3.2 Oxytocin, autonomic regulation and the HPA axis

Sexual response is also shaped by oxytocinergic signaling, autonomic regulation and HPA-axis activity (9, 50). These systems are particularly relevant to depression-related and antidepressant-associated sexual dysfunction because depression is associated with sustained stress-axis activation, altered autonomic balance and disturbed social-affiliative signaling (9, 51–53). Each system interacts with monoaminergic pathways, and together they help explain why sexual dysfunction in depression often involves desire, arousal, orgasmic quality and relational satisfaction rather than a single isolated domain (8, 9).

Oxytocin is synthesized mainly in the paraventricular and supraoptic nuclei of the hypothalamus and is released both centrally and peripherally (50). Centrally, oxytocin has been implicated in sexual arousal, erectile responses in men, genital arousal-related processes in women, orgasmic experience, pair bonding and post-coital affiliation, although human findings remain heterogeneous (50). Depression has been associated with altered oxytocinergic signaling, including changes in peripheral oxytocin levels and altered central responses to social or intimate stimuli (50). Reduced oxytocinergic responsiveness has been hypothesized to contribute to diminished intimacy, emotional connection and orgasmic satisfaction in depressed or antidepressant-treated patients, although direct causal evidence in AISD remains limited (50).

Autonomic regulation provides the peripheral output for sexual response (7, 20). Parasympathetic outflow supports genital engorgement, penile erection, clitoral and vaginal vasocongestion and lubrication, while sympathetic activity contributes to orgasm and ejaculation (7, 20). Depression and chronic stress are characterized by sympathetic predominance and parasympathetic withdrawal, often reflected in reduced heart rate variability (54–59). This imbalance can impair the parasympathetic activation required for genital arousal and vascular response, contributing to erectile difficulty in men and reduced lubrication or arousal in women independently of antidepressant medication (7, 57). However, HRV should be interpreted as a marker of autonomic regulation rather than as a direct biomarker of sexual function. Some antidepressants further modify autonomic tone, particularly tricyclic agents with anticholinergic activity, whereas SSRIs have more variable autonomic effects (35, 60).

HPA-axis activation adds a further mechanistic layer (9). Chronic stress and depression are associated with elevated corticotropin-releasing hormone, hypercortisolemia in a subset of patients and impaired negative feedback within the HPA axis (9, 61, 62). Sustained glucocorticoid exposure has been linked to reduced sexual interest, blunted arousal and impaired sexual function in both sexes through effects on reward circuits, endocrine signaling and vascular physiology (9, 38). The HPA axis also interacts bidirectionally with serotonergic and dopaminergic systems, so stress-related dysregulation may amplify the neurotransmitter imbalances that underlie both depression and AISD (8, 9). This convergence suggests that interventions targeting only a single neurotransmitter or a single vascular endpoint may produce incomplete clinical responses (16).

The integration of oxytocinergic, autonomic and HPA-axis biology with monoaminergic mechanisms provides a basis for examining plant-derived bioactives that affect more than one biological level (8, 9). Saffron, for example, has been studied for effects on mood symptoms, monoaminergic signaling and stress-related pathways (26, 63). Polyphenol-rich dietary patterns may influence oxidative stress, inflammatory tone, endothelial function and autonomic regulation in broader psychophysiological contexts (64, 65). Direct human evidence linking such nutritional modulation to improved sexual function in depressed or antidepressant-treated patients remains limited, but the mechanistic alignment is relevant to the evidence synthesis in Section 4 (16, 31).

### 3.3 Nitric oxide and vascular mechanisms

NO is a major mediator of the vascular events required for sexual response, and its bioavailability is sensitive to mood state, antidepressant exposure and dietary factors (20, 24). In men, neuronal and endothelial NO synthase isoforms promote penile smooth muscle relaxation and vasodilation during erection, while PDE5 hydrolyzes cyclic guanosine monophosphate to terminate the response (20, 24). In women, analogous NO-mediated mechanisms support clitoral and vaginal vasocongestion, lubrication and genital arousal, although the female vascular response has been less extensively characterized at the molecular level than male erection (7, 66). These shared molecular mechanisms position NO biology as an important substrate for sexual response across sexes and as an interface between nutrition and sexual function (7, 20).

Depression is increasingly recognized as being associated with vascular and endothelial dysfunction (21, 22). Patients with major depressive disorder show evidence of reduced flow-mediated dilation, increased arterial stiffness, elevated oxidative stress markers and altered circulating endothelial-related biomarkers, including NO-related and angiogenic markers, compared with non-depressed controls (21, 67–69). These vascular abnormalities partly reflect shared cardiometabolic risk factors, but they also appear to be shaped by neuroinflammation, sympathetic overactivity and HPA-axis dysregulation (70, 71). Reduced endothelial NO bioavailability provides a plausible mechanism linking depression to vascular components of sexual dysfunction, especially erectile difficulty in men and impaired genital arousal in women (20, 21, 72).

Antidepressant exposure may also influence vascular, autonomic and endothelial mechanisms, although effects differ across drug classes (53, 73). SSRIs have been associated with both pro- and anti-aggregatory platelet effects and with variable effects on endothelial function, while tricyclic antidepressants generally have less favorable cardiovascular profiles (73, 74). The peripheral vascular effects of SSRIs are usually less prominent than their central serotonergic effects on sexual function, but they may contribute to overall sexual response, particularly in patients with cardiometabolic risk (35, 73).

The vascular dimension is directly relevant to nutritional research because several plant-derived bioactives act on endothelial function and NO bioavailability (27, 28, 75). Flavonoid-rich foods, including berries, citrus fruits, apples, onions, cocoa and tea, have been associated with improved endothelial function, reduced arterial stiffness and lower cardiovascular risk in observational and intervention studies (64, 76). Dietary nitrates from beetroot and green leafy vegetables support NO signaling through the entero-salivary nitrate-nitrite-NO pathway and can improve endothelial function and blood pressure (27, 75, 77). Pomegranate polyphenols may enhance NO bioavailability by reducing oxidative inactivation of NO, and ginseng and ginsenosides may influence endothelial NO signaling, although most relevant evidence comes from erectile-function studies in men with mixed-etiology erectile dysfunction rather than from AISD cohorts (28, 34, 78).

These vascular mechanisms operate alongside central monoaminergic, oxytocinergic, autonomic and HPA-axis mechanisms (7, 9). Depression and antidepressant exposure can therefore affect sexual function at several biological levels simultaneously, from central motivation and affective engagement to peripheral vascular response (1, 21, 22). Plant-derived bioactives that engage one or more of these levels provide the mechanistic basis for the candidates discussed below (16, 31).

## 4 Plant-derived bioactives in depression-related and antidepressant-associated sexual dysfunction

The candidates examined in this section span two exposure scales: habitual dietary patterns and selected botanical preparations. Dietary patterns involve chronic exposure to multiple bioactives within food matrices, whereas botanical preparations differ in dose, matrix and degree of standardization (79). This distinction is important because mechanistic plausibility may transfer across these scales, but clinical evidence does not (31). Plant-derived bioactives comprise a chemically diverse group of food-derived and botanical compounds with potential relevance to depression-related and antidepressant-associated sexual dysfunction, but their relevance depends on more than biological plausibility (16, 32, 33). The key questions are whether a candidate engages pathways relevant to sexual response, whether its effects have been tested in the appropriate clinical phenotype, and whether safety, dose and formulation can be interpreted in patients receiving antidepressants (16, 80). This section first considers flavonoid-rich dietary patterns as a nutritional background, then evaluates selected candidates according to mechanistic engagement, human evidence and evidence boundaries.

### 4.1 Flavonoid-rich dietary patterns

Flavonoid-rich dietary patterns provide the nutritional context in which targeted botanical interventions should be interpreted (29, 81, 82). Unlike concentrated preparations, whole-food sources are consumed as part of habitual diets and affect several systems relevant to depression and sexual function, including endothelial NO signaling, oxidative stress, inflammatory tone, cardiometabolic risk and potentially neurovascular coupling (64, 81). Common sources include berries, citrus fruits, apples, onions, cocoa, tea and other plant foods that provide anthocyanins, flavan-3-ols, flavonols, flavones and flavanones.

Food matrices influence absorption, metabolism and biological response in ways that differ from purified extracts (79). Co-consumption with fats, fibers and other phytochemicals can alter intestinal uptake and post-absorptive metabolism, while gut microbiota transform polyphenols into metabolites that may mediate much of their systemic activity (79, 83). This microbiota-dependent dimension is relevant because individual differences in microbial composition may contribute to the heterogeneity observed across nutritional intervention studies (83). It also cautions against treating whole-food patterns and isolated botanical extracts as interchangeable interventions (79).

Prospective cohort studies have reported inverse associations between flavonoid intake and depression risk, particularly for anthocyanins and flavanones, although effect sizes are modest and confounding by overall diet quality and lifestyle remains difficult to exclude (84–87). At the vascular level, flavonoid-rich foods and healthful plant-based dietary patterns are associated with improved endothelial function, reduced arterial stiffness and favorable metabolic outcomes (30, 64). Plant-rich and Mediterranean-style dietary patterns combine flavonoid-rich foods with vegetables, legumes, whole grains, nuts and olive oil and have been investigated in relation to depressive symptoms and cardiometabolic health, although findings vary by population and dietary pattern (88–91). These patterns also support a less inflammation-prone gut microbiota and lower systemic inflammatory tone, both of which are relevant to mood regulation and endothelial physiology (92, 93).

The relevance of flavonoid-rich dietary patterns to depression-related and antidepressant-associated sexual dysfunction is therefore indirect but mechanistically coherent (29, 64). They may support vascular and inflammatory conditions that influence sexual response, and they may contribute to broader mood-related physiology (21, 81). Current AISD intervention reviews have not identified clinical trials testing flavonoid-rich dietary patterns for AISD (16, 94). Flavonoid-rich dietary patterns should therefore be framed as a background strategy that supports relevant biological systems, while existing AISD intervention reviews do not establish them as AISD-specific interventions (16).

### 4.2 Saffron (*Crocus sativus*)

Saffron, derived from the dried stigmas of *Crocus sativus*, is the plant-derived candidate with the most coherent AISD-specific clinical signal (25, 32, 95). Its principal constituents include crocin, crocetin, safranal and picrocrocin, which have been studied for antioxidant, anti-inflammatory and neuromodulatory effects (96). Mechanistically, saffron has been linked to serotonergic signaling, monoamine oxidase activity, oxidative stress, inflammatory tone and interactions with dopaminergic and glutamatergic pathways (26, 97). These mechanisms map onto several biological systems implicated in depression-related and antidepressant-associated sexual dysfunction (32, 95).

The broader antidepressant literature provides an important context for interpreting saffron in AISD (63, 98). Small randomized trials and meta-analyses suggest that saffron, commonly administered at 30 mg/day for 6–8 weeks, may reduce depressive symptoms (63, 99). Some head-to-head trials have reported symptom reductions comparable to standard antidepressants such as fluoxetine or imipramine, although most studies were limited by small sample size and short duration (100). This mood-related evidence does not establish efficacy for sexual dysfunction, but it suggests that saffron acts on pathways relevant to the depressive component of the phenotype (26).

AISD-specific evidence comes from two small randomized, double-blind, placebo-controlled trials in patients with fluoxetine-associated sexual dysfunction (32, 95). In women with major depressive disorder whose depression had remitted during fluoxetine treatment, adjunctive saffron 30 mg/day for 4 weeks improved the total Female Sexual Function Index score and specific domains including arousal, lubrication and pain, while desire, orgasm and satisfaction were less consistently affected (32). In men with fluoxetine-associated sexual dysfunction, adjunctive saffron 30 mg/day for 4 weeks improved erectile function and intercourse satisfaction domains of the International Index of Erectile Function, while orgasmic function, sexual desire and overall satisfaction did not differ significantly from placebo (95).

This domain-specific pattern is important (32, 95). Saffron did not act as a non-specific sexual enhancer in these trials (32, 95). Its apparent effects were concentrated in arousal- and genital-response-related domains, a pattern compatible with, but not proving, multi-pathway mechanisms involving mood, oxidative stress, endothelial physiology and central signaling (32, 95). The evidence base remains limited by trial number, sample size, short duration and the absence of direct comparison with bupropion or PDE5 inhibitors (16, 25). Nevertheless, among the candidates reviewed here, saffron currently provides one of the clearest phenotype-specific proof-of-concept signals for further study in AISD (25, 32, 95). Across these trials, the reported benefits and tolerability were observed at a single, consistent studied dose of 30 mg/day; this dose can therefore serve as a reasonable reference dose for future comparative trials, while recognizing that its status as an optimal or clinically confirmed effective dose remains to be established in larger studies.

### 4.3 Maca (*Lepidium meyenii*)

Maca is a cruciferous root traditionally cultivated in the Andean highlands (101, 102). Maca preparations contain glucosinolates, macamides, macaenes, sterols and other bioactives that have been studied in relation to sexual function, mood and energy (102). Its relevance to depression-related and antidepressant-associated sexual dysfunction lies primarily in potential non-hormonal effects on sexual desire, energy and central motivational pathways rather than in direct gonadal endocrine stimulation (33, 103).

The most directly relevant clinical study is a double-blind, placebo-controlled trial of maca root in women with SSRI- or SNRI-associated sexual dysfunction whose depression had remitted (33). Participants received maca 3.0 g/day for 12 weeks, and sexual function was assessed using the Arizona Sexual Experience Scale and the Massachusetts General Hospital Sexual Function Questionnaire (33). Treatment signals favored maca over placebo, with the clearest exploratory signal observed in postmenopausal women (33). The study was modest in size, but the postmenopausal signal is clinically informative because it suggests a phenotype in which central motivational modulation may interact with menopausal endocrine context (33).

Earlier randomized trials in healthy adult men reported improvements in self-reported sexual desire after maca supplementation at 1.5–3.0 g/day for 8–12 weeks (103). These studies did not show changes in testosterone or estradiol, supporting the interpretation that maca’s effects on desire are not mediated by gonadal hormone elevation (103). Limited additional data in men with mild erectile difficulties suggest modest improvements, but the evidence is not sufficient to infer efficacy in male AISD (102, 104). Maca should therefore not be characterized as a testosterone-boosting intervention (103). Its current evidence supports a narrower interpretation: a candidate dietary modulator with limited but coherent evidence in female AISD, particularly in postmenopausal women, and supplementary evidence for non-hormonal effects on male desire (33, 103). The clearer signal in postmenopausal women is mechanistically plausible. In this group, reduced ovarian estrogen lowers baseline genital vasocongestion and desire, so a candidate acting mainly on central motivational and non-hormonal pathways, as maca appears to, may produce a more detectable improvement than in premenopausal women whose endogenous hormonal milieu is largely intact. This also indicates that male and female responses to the same compound should not be assumed equivalent, because the hormonal and vascular context in which the compound acts differs by sex.

### 4.4 Rosa damascena and *Ginkgo biloba*

Rosa damascena and *Ginkgo biloba* illustrate two different evidence patterns in the botanical AISD literature (105–107). Rosa damascena has preliminary trial evidence with modest effects, while *Ginkgo biloba* has attracted sustained clinical interest but has produced inconsistent controlled findings (106–108). Both candidates remain secondary to saffron and maca in the current evidence hierarchy (16, 25).

Rosa damascena, traditionally known as Damask rose, has been used as an aromatic and medicinal plant (106). Its extracts and essential oil contain phenolic compounds, terpenes and flavonoids, and it has been proposed to exert anxiolytic, antioxidant and neuromodulatory effects (106). These mechanisms remain less directly characterized in AISD than those proposed for saffron, and any relevance to sexual function should be interpreted cautiously (106, 107). Small randomized trials of Rosa damascena oil or extract preparations in patients with SSRI-associated sexual dysfunction reported improvements in selected sexual-function parameters in men and more modest effects in women (106, 107). The evidence remains preliminary, with variability in preparation, dose and outcome assessment (106, 107). A recent systematic review of pharmacological treatment for antidepressant-associated sexual dysfunction in women included Rosa damascena but concluded that effects were limited and inconsistent within an overall low-certainty evidence base (16).

*Ginkgo biloba* extract, particularly standardized EGb 761, has been examined for cognitive, vascular and neuropsychiatric indications, including SSRI-associated sexual dysfunction (105, 108). Its principal constituents include flavonoid glycosides and terpene lactones such as ginkgolides and bilobalide, which may influence endothelial NO signaling, oxidative stress and platelet function (108, 109). Early open-label observations in the 1990s suggested improvement in antidepressant-associated sexual dysfunction across several domains in both sexes (110). Subsequent randomized, placebo-controlled evidence is best interpreted as negative or inconsistent: the dedicated placebo-controlled trial in antidepressant-associated sexual dysfunction did not show superiority over placebo, and the broader clinical trial literature reports variable, mostly modest or domain-specific findings without consistent statistically significant effects (105, 108). *Ginkgo biloba* also carries a potential bleeding-related interaction concern when combined with anticoagulant or antiplatelet medications (109, 111, 112). Its inclusion in this review therefore reflects historical prominence and ongoing research interest rather than strong contemporary evidence (105, 108).

### 4.5 *Panax ginseng*, pomegranate and dietary nitrates

*Panax ginseng*, pomegranate and dietary nitrates share a common mechanistic theme: each can engage NO and endothelial biology, which is relevant to the vascular component of sexual response (34, 75, 78). None has been adequately tested in AISD-specific cohorts (16, 34, 75, 78). Their relevance to depression-related and antidepressant-associated sexual dysfunction is therefore inferred from adjacent populations, including men with mixed-etiology erectile dysfunction, adults in cardiovascular studies and postmenopausal women with general sexual concerns (28, 34, 78).

*Panax ginseng* and its ginsenosides have been examined for effects on fatigue, immune regulation and sexual function, with the most consolidated evidence relating to erectile function in men (78, 113). A recent food-medicine homology review further summarizes ginseng’s edible and medicinal value, bioactive ingredients, processing approaches and product forms (114). A Cochrane review and several randomized controlled trials suggest that ginseng may produce small improvements in erectile function compared with placebo, although certainty is low and effects vary across studies (78). Proposed mechanisms include central catecholaminergic modulation, endothelial NO synthase activity and antioxidant effects (115, 116). However, most trials enrolled men with erectile dysfunction of mixed etiology rather than depressed or antidepressant-treated patients (78). Evidence in postmenopausal women with sexual concerns is limited and suggests at most modest improvements in arousal (117, 118). Ginseng is therefore relevant mainly as a vascular and central neuromodulatory candidate requiring phenotype-specific testing (78).

Pomegranate (*Punica granatum*) contains punicalagins, ellagic acid and anthocyanins that may enhance NO bioavailability by reducing oxidative inactivation of NO (28). Recent pharmacological work on ellagic acid and urolithins further supports their relevance to metabolic and antioxidant pathways, but this evidence remains adjacent rather than AISD-specific (119). A randomized, placebo-controlled crossover study of pomegranate juice in men with mild to moderate erectile dysfunction reported a favorable but statistically non-significant trend compared with placebo (34). This finding should therefore be treated as adjacent erectile-dysfunction evidence rather than evidence of efficacy in AISD, and the small sample size and mixed etiology further limit inference for depression-related or antidepressant-associated sexual dysfunction (28, 34).

Dietary nitrates from beetroot, leafy greens and other vegetables support NO signaling through the entero-salivary nitrate-nitrite-NO pathway (27, 75). Acute and short-term nitrate supplementation can improve endothelial function, lower blood pressure and enhance exercise tolerance in adults, although effects vary across populations and intervention durations (27, 75, 120). Direct trials of dietary nitrates in sexual function, particularly in depression-related or antidepressant-associated sexual dysfunction, are essentially absent (16, 75). This absence is notable because nitrate biology maps clearly onto the vascular component of sexual response (27, 75, 121). For now, dietary nitrates should be considered mechanism-driven candidates rather than evidence-based interventions for AISD (27, 75). This also has a practical implication for study design: because dietary nitrate from foods such as beetroot is difficult to quantify precisely in free-living dietary settings, trials intended to test a nitrate-based hypothesis in AISD should define exposure rigorously, whether through standardized nitrate supplements or tightly controlled nitrate-rich dietary protocols, so that any effect can be tied to a reproducible intake.

### 4.6 Cross-candidate synthesis

The candidate evidence base is uneven in both strength and phenotype specificity. Flavonoid-rich dietary patterns provide a biologically coherent nutritional background through effects on mood, vascular function, inflammation and cardiometabolic health, but no AISD-specific trials have tested their clinical effect (29, 30, 122). Saffron has the most coherent direct evidence, supported by parallel small randomized trials in women and men with fluoxetine-associated sexual dysfunction and by a broader antidepressant literature (25, 32, 95). Maca has a narrower signal in female AISD, particularly in postmenopausal women, and supplementary evidence for non-hormonal effects on male desire (33, 103). Rosa damascena remains preliminary and modest (106, 107). *Ginkgo biloba* remains historically important but inconsistently supported. *Panax ginseng*, pomegranate and dietary nitrates have plausible vascular mechanisms but lack direct AISD evidence (28, 34, 78). Overall, the mechanistic rationale is coherent, whereas the clinical evidence remains limited and uneven (16, 31).

A second pattern concerns sex-specific evidence. Female AISD has been the focus of several randomized trials using validated instruments such as the Female Sexual Function Index, Arizona Sexual Experience Scale and Massachusetts General Hospital Sexual Function Questionnaire (32, 33, 123). Within this phenotype, saffron appears to affect arousal, lubrication and pain in women with fluoxetine-associated sexual dysfunction, while maca may improve sexual function most clearly in postmenopausal women with SSRI- or SNRI-associated sexual dysfunction (32, 33). A systematic review of pharmacological treatment for antidepressant-associated sexual dysfunction in women included plant-derived candidates alongside bupropion, sildenafil and testosterone, and identified saffron and maca as preliminary candidates requiring further investigation within a generally low-certainty evidence base (16).

The male evidence base is structurally different. Dedicated male AISD trials remain sparse, and much of the broader male literature concerns erectile dysfunction of mixed etiology assessed with the International Index of Erectile Function or related instruments (34, 78). This creates two interpretive problems. First, evidence for ginseng, pomegranate and vascular nutritional interventions informs erectile physiology but is not phenotype-specific to AISD (34, 78). Second, maca evidence in men addresses sexual desire in healthy adults rather than antidepressant-associated dysfunction (103). The clearest exception is saffron, for which a small trial in men with fluoxetine-associated sexual dysfunction provides direct but limited proof-of-concept evidence concentrated in erectile function and intercourse satisfaction (95).

A third pattern concerns the distinction between depression-related and antidepressant-associated sexual dysfunction. Many patients with major depressive disorder experience sexual dysfunction before pharmacotherapy begins, and depression is also associated with disturbances in reward processing, autonomic balance, HPA-axis activity, oxytocinergic signaling and endothelial function (2, 9, 21). Most trials of plant-derived candidates have enrolled patients already receiving antidepressants, which limits attribution of improvement to depression-related versus medication-related mechanisms (32, 33, 95). Candidates with both antidepressant and sexual-function signals, especially saffron, are mechanistically plausible in this phenotype, but that role remains a testable hypothesis rather than an established clinical indication (26, 32, 95).

Taken together, the evidence supports a tiered interpretation. Saffron is the most coherent candidate for further AISD-specific investigation in both sexes (32, 95). Maca is a focused candidate for female AISD, especially in postmenopausal women (33). Rosa damascena may be considered preliminary, while *Ginkgo biloba* requires cautious interpretation because of inconsistent efficacy and interaction risk (105, 106, 109). *Panax ginseng*, pomegranate and dietary nitrates remain mechanism-driven candidates whose relevance is currently indirect (28, 34, 78). None has reached the level of evidence required for routine clinical recommendation (16). Their most appropriate current framing is adjunctive nutritional modulation within evidence-based psychiatric and sexual-medicine care (16). The mechanistic engagement, direct clinical evidence, typical dosing and safety of each candidate are summarized in Table 1. Preclinical botanical studies without antidepressant-treated populations or sexual-function outcomes were treated as outside the direct AISD evidence base, even when they addressed adjacent neuropsychiatric mechanisms (124).

**Table 1:** Plant-derived bioactives in depression-related and antidepressant-associated sexual dysfunction.

Candidate	Mechanistic engagement	Direct AISD evidence (sex)	Typical dose	Preparation / standardization	Evidence category	Safety / interaction
Flavonoid-rich dietary patterns	Endothelial NO; oxidative and inflammatory tone; cardiometabolic	No AISD trials; inverse association with depression risk	Dietary	Whole-diet exposure; not standardized	Background dietary strategy	Generally safe as food
Saffron (*Crocus sativus*)	Serotonergic and MAO activity; antioxidant, anti-inflammatory	RCTs in women and men with fluoxetine-associated SD (women: arousal, lubrication, pain; men: erectile function, satisfaction)	30 mg/day, 4 wk	Standardized extract; crocin/safranal	Direct AISD evidence (positive)	Well tolerated at 30 mg/day; theoretical serotonergic caution at high dose
Maca (*Lepidium meyenii*)	Central motivational, non-hormonal	RCT in women with SSRI/SNRI-associated SD; clearest in postmenopausal women; male data on desire only	1.5–3.0 g/day, 8–12 wk	Powder or extract; macamide content varies	Direct AISD evidence (positive, female)	Well tolerated; effect not testosterone-mediated
Rosa damascena	Anxiolytic, antioxidant, neuromodulatory	Small RCTs in SSRI-associated SD; modest, greater in men	Oil/extract, variable	Oil or extract; poorly standardized	Preliminary AISD evidence	Generally regarded as safe; preparation varies
*Ginkgo biloba*	Endothelial NO, platelet function, antioxidant	Dedicated RCT negative; overall inconsistent	EGb 761, variable	EGb 761 standardized extract	Direct AISD evidence (null)	Potential bleeding-related interaction concern with anticoagulant/antiplatelet therapy
*Panax ginseng*	Central catecholaminergic; eNOS; antioxidant	ED trials of mixed etiology; limited female arousal data; no AISD trial	Variable	Extract; ginsenoside content varies	Adjacent sexual-function evidence	Warfarin interaction; metabolic considerations
Pomegranate	Enhances NO bioavailability via antioxidant effect	ED crossover trend (non-significant); no AISD trial	Juice/extract	Juice or extract; polyphenol content varies	Adjacent sexual-function evidence	Generally safe as food
Dietary nitrates	Entero-salivary nitrate-nitrite-NO; endothelial	No sexual-function trials; vascular effects only	Dietary/supplement	Food (beetroot) vs. supplement; hard to quantify	Mechanistic only	Safe as food; dose hard to quantify

## 5 Limitations, safety and future directions

### 5.1 Clinical and methodological limitations

The clinical evidence base for plant-derived bioactives in AISD shares several limitations common to research on plant-derived preparations (16, 80). Sample sizes are generally small, with most trials enrolling fewer than 100 participants (32, 33, 95). Trial durations are short, usually 4–12 weeks, which limits assessment of sustained efficacy, relapse effects and long-term safety (32, 95). Outcome instruments vary across studies, complicating direct comparison and meta-analysis (16, 31). Standardization of plant-derived preparations is often incomplete, with differences in extraction methods, bioactive content and batch-to-batch variability that affect reproducibility (80, 125, 126). Blinding can be difficult because of the sensory properties of botanical preparations, yet blinding adequacy is rarely examined in detail (80, 125). The degree of standardization also differs by candidate; for example, crocin content in saffron extracts and macamide content in maca preparations vary between products, which further limits reproducibility. In addition, because most trials lasted only 4 to 12 weeks, there are currently no data on long-term tolerability or on discontinuation effects after prolonged supplement use, which constrains conclusions about sustained safety. Finally, because the principal outcomes were expectancy-sensitive self-report scores such as the FSFI and IIEF, a placebo contribution to the observed improvements cannot be excluded; future studies should include adequately powered placebo-controlled designs and, where feasible, active-comparator arms (for example bupropion), to distinguish placebo response from candidate-specific effects (80, 122).

Three AISD-specific limitations are particularly important (127). First, sex-specific evidence remains uneven. Female AISD trials currently provide the most direct data for saffron and maca, while male AISD evidence is limited largely to one saffron trial and indirect erectile-function studies (32, 33, 95, 128). Second, depression-related and antidepressant-associated phenotypes are frequently conflated (2). Most trials enroll patients already receiving antidepressants with persistent sexual symptoms, which limits mechanistic attribution and excludes the pre-treatment depression-related phenotype (1, 127). Third, plant-derived candidates have rarely been compared directly with established adjunctive strategies (16, 17). For saffron, a head-to-head comparison with bupropion is especially important because saffron has both antidepressant and AISD-specific signals, while bupropion remains an empirical benchmark for adjunctive pharmacological management (17, 32, 95). Because bupropion is among the most consistently supported pharmacological options for AISD and is widely used as a clinical comparator, a direct saffron-versus-bupropion comparison would be especially informative.

### 5.2 Safety and interaction considerations

Safety considerations are central when plant-derived interventions are used alongside antidepressant therapy. Saffron at commonly studied clinical doses, typically 30 mg/day, appears generally well tolerated, but high-dose exposure or combination with multiple serotonergic agents raises theoretical concerns about serotonin toxicity that require clinical caution (63). Maca is generally well tolerated at typical doses of 1.5–3.0 g/day, with few serious adverse effects reported in trials (33, 103). Rosa damascena preparations are generally regarded as safe, although product standardization varies (106, 107). *Ginkgo biloba* carries a potential bleeding-related interaction concern when combined with anticoagulant or antiplatelet medications (109, 112). *Panax ginseng* may also have medication-interaction and metabolic considerations in selected clinical contexts (129, 130). Beyond coagulation-related concerns, pharmacokinetic interactions should be considered because antidepressants are commonly metabolized through CYP2D6, CYP2C19, CYP3A4 or CYP1A2 pathways, and some botanical preparations may modulate cytochrome P450 enzymes or drug transporters depending on extract composition, dose and duration. The clinical relevance of these interactions remains preparation-specific and should not be assumed without pharmacokinetic evaluation (126, 130, 131). The tolerability of saffron at the 30 mg/day dose used in the trials reviewed here is also supported by broader safety and health-outcome reviews (96, 97).

These considerations shape candidate selection. A patient with prominent vascular comorbidity may be biologically relevant to NO-targeted interventions but may also be taking antiplatelet or anticoagulant therapy, making *Ginkgo biloba* less appropriate (109, 112). A patient with unstable depression may not be suitable for antidepressant dose reduction or drug holidays, but could be considered for adjunctive strategies only within careful psychiatric follow-up (1, 12, 18). Plant-derived bioactives should therefore not be interpreted as stand-alone treatments (16). Their use requires attention to depressive symptom status, antidepressant regimen, sexual-function profile, comorbidities, pregnancy considerations and concurrent medication exposure (16, 130, 131).

### 5.3 Dietary context and future trial design

Plant-derived candidates should be interpreted within a broader dietary and lifestyle context rather than as isolated agents. Plant-rich and Mediterranean-style dietary patterns have been investigated in depressive disorders, with evidence suggesting possible mood and cardiometabolic relevance, although findings vary by population and dietary pattern (29, 30, 132). These patterns engage the same biological substrates disturbed in depression and AISD, and they may be more acceptable to patients than interventions framed explicitly around sexual dysfunction (21, 29). However, botanical trials often provide limited contextual characterization, and baseline diet quality, physical activity, sleep and metabolic status may modify treatment response (16, 80). From a practical standpoint, this supports optimizing overall diet quality and lifestyle, including a Mediterranean-style dietary pattern, as a first-line background measure before targeted supplementation is considered.

Future AISD trials should document baseline diet quality, anthropometric and metabolic indices, medication history, depression severity, relationship factors and key lifestyle variables (80, 125). This would allow assessment of effect modification and reduce confounding from unmeasured behavioral factors (80, 125). Mechanistic endpoints should be selected according to candidate biology rather than added generically (80, 125). Saffron trials should assess mood, arousal domains and relevant stress or monoaminergic proxies where feasible (32, 95). Maca trials should stratify by menopausal status and assess desire-related outcomes (33, 103). NO-targeted candidates should include vascular endpoints such as flow-mediated dilation, blood pressure and NO metabolites (21, 27, 120).

The immediate priority is not simply to expand the list of candidates, but to test the most plausible existing candidates in phenotype-specific, adequately powered trials (32, 33, 95). A high-priority next trial would be a randomized, adequately powered comparison of saffron with bupropion and placebo in patients with SSRI-associated sexual dysfunction, stratified by sex and menopausal status (17, 32, 95). Such a design would directly address comparative effectiveness, phenotype specificity and mechanistic interpretation (16, 17). Until such data are available, plant-derived bioactives should be considered investigational adjunctive modulators rather than established interventions for depression-related or antidepressant-associated sexual dysfunction (16, 31).

## 6 Conclusion

Depression-related and antidepressant-associated sexual dysfunction is a multidimensional clinical problem in which depressive illness, antidepressant exposure and vascular or endocrine physiology can each contribute to sexual symptoms (1, 2, 21). This multidimensionality helps explain why symptoms often span desire, arousal, orgasm, genital response and satisfaction, and why single-target interventions provide only partial benefit for many patients (16, 31). Plant-derived bioactives are relevant to this field because several candidates engage biological systems implicated in sexual response, including monoaminergic signaling, inflammatory and oxidative pathways, HPA-axis activity, autonomic balance and NO-mediated vascular function (27, 32, 95).

Saffron has the clearest direct signal among the candidates reviewed, with small randomized trials in women and men with fluoxetine-associated sexual dysfunction and broader evidence for antidepressant activity (32, 95). Maca provides a narrower signal in female AISD, particularly among postmenopausal women, and supplementary evidence for non-hormonal effects on sexual desire in men (33, 103). Rosa damascena remains preliminary, *Ginkgo biloba* remains inconsistent and safety-limited, and *Panax ginseng*, pomegranate and dietary nitrates remain supported mainly by vascular plausibility and adjacent clinical evidence (78, 105, 106).

Future progress will depend on moving from candidate identification toward phenotype-specific evidence. Larger and longer randomized trials, standardized preparations, sex-specific analyses, validated sexual-function instruments, mechanistic biomarkers and careful interaction assessment are needed (16, 80). Direct comparison with established adjunctive strategies, especially bupropion, will be essential for defining clinical relevance (16, 17). The immediate priority is rigorous testing of existing plausible candidates rather than the discovery of new ones (32, 33, 95). Until then, plant-derived bioactives should be positioned as investigational adjunctive modulators within integrated psychiatric, sexual-medicine and nutritional care, rather than as replacements for established treatment (16, 31).
